# From “One Health” to “One Communication”: The Contribution of Communication in Veterinary Medicine to Public Health

**DOI:** 10.3390/vetsci2030135

**Published:** 2015-07-15

**Authors:** Micaela Cipolla, Luigi Bonizzi, Alfonso Zecconi

**Affiliations:** Department of Veterinary Science and Public Health (DIVET), Università degli Studi di Milano, via Celoria 10, 20133 Milano, Italy; E-Mails: luigi.bonizzi@unimi.it (L.B.); alfonso.zecconi@unimi.it (A.Z.)

**Keywords:** communication, One Health, public health, antimicrobial resistance, food safety, food-producing animals, risk communication, companion animal practice

## Abstract

Despite the fact that health communication is a discipline developed only recently, its importance in human medicine is well recognized. However, it is less considered in veterinary medicine, even if it has the potential to improve public health because of the role of veterinary medicine in public health. For this reason, an One Health approach is useful for communication as well. This approach leads to a “One Communication” concept, which is the result of the synergy in communicative efforts both in human and in veterinary medicine. Our analysis explores the potential of communication in several veterinary fields: institutions, food safety, companion animal and food-producing animal practice, pharmacology and drugs, wildlife fauna and environment. In almost all the areas of veterinary activity communication can contribute to human health. It takes many forms and use several channels, and this variety of communicative opportunities represent a challenge for veterinarians. For this reason, the communication course should be included in the curricula of Veterinary Medicine Schools. As One Health, One Communication is a strategy for expanding collaborations in health communication and it will enhance public health.

## 1. Introduction

Health Communication is a discipline only recently developed, and it is “The study of how health information is generated and disseminated and how that information affects individuals, community groups, institutions and public policy” [1].

The aim of health communication is to inform and influence people’s behaviour and attitude in order to improve health [2,3,4]. This is possible because it concerns all aspects of health, including, *i.e.*, research, clinical practice, public health, global health and policy making [5,6]. Different approaches are available to investigate all of the aspects, *i.e.*, health campaigns, risk communication and patient-provider communication.

An effective communication is essential to public health. It can be written and verbal, taking many forms and using various channels such as physicians and other health professionals, family, friends and mass media, Internet, advertisement. For its growing importance, the World Health Organization (WHO) includes health communication in its activities and provides several guides supporting communication strategies development for different situations, such as health emergencies and disease prevention [7,8,9,10,11,12,13]. Moreover, WHO considers communication expertise as essential to outbreak control as well as epidemiological training and laboratory analysis, and demonstrated a significant reduction of cases during an outbreak when applied a proactive communication [14,15]. Many other institutions, such as UNICEF, FAO, CDC, Johns Hopkins University and others agree that communication is a necessary tool for public health [16,17,18,19,20,21,22,23,24,25,26,27,28].

Any advice aiming to enhance health by changing people’s behaviour is a communicative act. Health communication allows to identify contexts, channels, messages and factors having the potential to motivate individuals to use correctly health information. Therefore, health communication supports health professionals in their daily work, educates patients and helps policy makers; it has a powerful role in disease prevention and health promotion. It is part of health management, even in settings with limited resources, helping to use information and interacting with the community or other partners.

Due to its pivotal importance, health communication became part of the curriculum in several Medicine and Veterinary Medicine Schools worldwide, but usually health communication refers to human health and it is generally less considered in veterinary medicine. The intended outcome of this article is to describe this new aspect of veterinary medicine—the health communication—and its contribution to public health. With our paper we highlight the role of communication in the main branches of veterinary activity, to help veterinarians to find their role in the “One communication” perspective. Indeed, communication in veterinary medicine has the potential to contribute to public health and should be considered in a One Health perspective.

### One Health

The One Health concept links human, animal and environmental health, it is “a worldwide strategy for expanding interdisciplinary collaborations and communications in all aspects of health care for humans, animals and the environment. The synergism achieved will advance health care for the 21st century and beyond by accelerating biomedical research discoveries, enhancing public health efficacy, expeditiously expanding the scientific knowledge base, and improving medical education and clinical care.”[29]. This approach considers all global health threats to gain cross-disciplinary collaboration, especially between physicians and veterinarians. The result is a collaborative effort to enhance human and animal health between the health professions.

One Health has its origins in the 1800s, when Rudolf Virchow, the German physician who coined the term "zoonosis", highlighted the linkages between human and veterinary medicine [30]. In the following years, other physicians and veterinarians recognized the importance of good animal health for the public health. One of them, Calvin Schwabe, made important contributions to epidemiology, zoonoses, tropical health and public health. He coined the term “One medicine”, emphasizing the close interdependence and similarities between human and veterinary medicine [31]. More recently, the Wildlife Conservation Society promoted a symposium to discuss the movements of diseases among human, domestic animal and wildlife populations. The symposium set twelve priorities—the Manhattan Principles—for an interdisciplinary strategy to combat diseases and maintain the ecosystem integrity. These principles considered, with human and animal health, also environment health [32].

The One Health approach has been endorsed by the main global health organizations, such as WHO, CDC, EFSA, OIE, FAO [33,34,35,36,37]. The collaborative effort between human and veterinary medicine should be applied not only at global level but also at local level, where it could be a new paradigm of health management [38]. This will result in a more effective and sustainable organization of public health, reducing the risk of zoonotic diseases such as rabies, H5N1 avian influenza, West Nile disease, TBC and diseases caused by E. coli STEC, *Staphylococcus aureus* MRSA, Nipah and Hendra viruses, Hantaviruses, Filovirus such as Ebola and Marburg viruses.

## 2. Veterinary Medicine and Public Health

**Figure 1:**
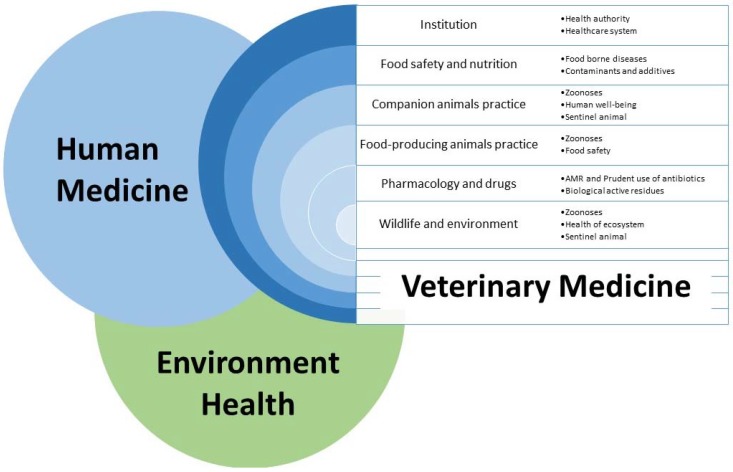
Potential contributes of veterinary medicine to public health in a One Health and One Communication perspective.

The purpose of veterinary medicine is to protect public health, through the promotion of animal health and food safety. To fulfil this objective, veterinary medicine has many arenas of activity in public health, including (Figure 1):

- Institutions (health authority, healthcare system)
- Food safety and nutrition
- Clinical practice
  ○ companion animals
  ○ food-producing animals
- Pharmacology and drugs
- Wildlife fauna
- Environment

In all these fields, communication has a key role and can contribute to public health by influencing people’s behaviour and attitude.

### 2.1. Institutions

Health care authorities and systems have the responsibility to manage health system, and communication is part of the management. Therefore, the role of communication is related to the institutional setting. We reported, as example, one European health system. In the Italian health system, communication for human and veterinary medicine is unique at the higher institutional level, because the Ministry of Health (Ministero della Salute) manages both. At lower regional and local institutional level, health authorities work separately on human and animal health, so the communication efforts often are uncoordinated. However, the institutional communication in veterinary medicine gives a high contribution to public health, because of the continuous overlapping between veterinary and human medicine in public health. This institutional activity includes, for example, health campaigns on zoonoses or food safety [39,40].

This organization of veterinary system has both pros and cons. The major benefit of the integration in the Ministry of Health is that the veterinary health system has a peculiar focus on health, and it is much less biased by production aspects, when compared to the systems under the Ministry of Agriculture. However, the focus on health, sometimes, put an excessive pressure on the production system, reducing its efficiency, by applying unnecessary barriers to the production process. At the local level, the system splits into the two components of human and veterinary medicine, and this disconnection gives to the veterinary medicine the opportunity to be more independent and effective in a given area, even if a coordination would be hopeful.

Moreover, the veterinary diseases surveillance networks have a key role in human surveillance and its utility would be increased through a better integration between the two systems, as happens through the joining of health service [41]. Likewise, a coordination also at the communicative level would produce more efficient efforts in enhancing public health. Also at policy and management levels, communication has a key role, as demonstrated by the example of Regional Veterinary Prevention Plan 2012–2014 (Piano Regionale della Prevenzione Veterinaria 2012–2014) of Regione Lombardia [42]. This was a new and powerful tool for public health, supporting veterinary health system at regional level, giving to all the operative units and officers an unique and coherent frame of reference [43]. Indeed, our research group developed a model (scorecard) to assess zoonoses and food safety issues. The scorecard supports daily practice setting priority in prevention and risk management, by assigning different scores to various risk and management aspects of each disease or food safety problem. Once this assignation has been done, the veterinary officers know the priority of each intervention and, also, if there is a lack of information about the specific disease. This model established a completely new approach to risk characterization in Italian public health authorities; it had to be understood and adopted by all the personnel involved in the regional veterinary system and, for this reason, communication was crucial. Both internal and external communication strategies were needed. All the members of the regional healthcare system were the audiences of internal communication: Regione Lombardia, local health authorities (Azienda Sanitaria Locale), regional diagnostic and surveillance laboratory (Istituto Zooprofilattico Sperimentale della Lombardia e dell’Emilia Romagna), research centers. At internal level, the aims of communication were to verify the outcomes of the plan application, to share information and to consolidate cooperation. External communication was addressed to stakeholders, with the aims to involve people, gather information and share results. All the initiatives, planned to inform and involve the target audience, were focused on objectives and results of the prevention plan application. However, the first communication need concerned the plan itself, and the understanding of the new risk characterization praxis. Indeed, the document was very articulated; it had many tables (the scorecards) and schemes for disease control plans, prevention protocols and risk characterization, each one concerning one of the veterinary areas. Indeed, in Italy the veterinary health system has three areas: A for animal health, B for food safety and C for herd management and animal welfare. One of the parameters considered for an effective communication of the plan was its graphic aspect. Due to the high amount and complexity of information delivered, we chose a simple and immediate graphic aspect, assigning a specific colour to each area. Every piece of information addressed to the animal health sector was green, to the food safety sector was red and to the herd management and animal welfare sector was yellow. Everything concerning the past or general topics was blue. This simple color code had a very useful function clarifying the whole document and making it more understandable. Moreover, this code made every local health authority having the same color for the same unit. All the veterinary officers recognized themselves in the color of their area, independently by the district of the local health authority. Therefore, the primary aim of the plan—to make uniform veterinary health system to improve public health—was achieved also with this simple communicative choice, and it was maintained also in the subsequent edition of the plan [44].

### 2.2. Food Safety and Nutrition

Communication on food safety and nutrition frequently pertains to veterinary medicine, being related to food-borne disease, GMO, contaminants, additives and foods of animal origin.

Consumers receive much information about food, but often it is contradictory and conflicting. This includes alerts, recommendation on what is good for health and what could be dangerous, risks and benefits associate in the same food, food components to reduce, nutrients to increase, warning on different diets and eating styles. All this information provide a non-unique frame of reference, which is difficult to manage and to understand by consumers. Sometimes unfounded concerns may overlap reliable messages from health authorities, resulting in more confusion and uncertainty [45]; especially during an alert or an outbreak, this situation may lead to fear and distrust in the product involved. Examples are the recent European food scares on *E. coli* in vegetables, horsemeat scandal, blue mozzarella cheese, BSE and avian influenza.

The health and welfare of food-producing animals during breeding, transportation and slaughtering have consequences for human health and are responsibility of veterinarians. Therefore, due to the pivotal role of veterinary medicine in animal nutrition, animal health, food inspection and food safety, communication in this area is important for public health, contributing to consumers’ protection from field to fork.

Communication about food is often risk communication. Accordingly with the Codex Alimentarius Commission (CAC), risk communication is “an interactive exchange of information and opinions concerning risk among risk assessors, risk managers, consumers and other interested parties [46]. Therefore, food risk communication enables informed decision-making to promote health, fosters the understanding of both the real and the perceived food risk, and shares knowledge between government officials, health professionals, academicians, food producers, stakeholders, journalists and public. Misleading messages can have serious consequences not only on public health but also on the entire food supply chain. Thanks to a proper communication, authorities, experts and other stakeholders can communicate more successfully, making the public reacting properly [47].

To meet these needs, many local and international organizations, such as EFSA and FAO, promoted food risk communication [48,49].

### 2.3. Clinical Practice

#### 2.3.1. Companion Animals

Companion animals, due to their close relationship with people, are important in the prevention of zoonoses and in their management. Moreover, pets have a role as sentinel animal and could be used to protect human health. For example, they are useful in cancer prevention and treatment in humans, because often they share the same risk and carcinogen factors, have many of the same types of cancer and are treated almost with the same drugs as humans [50]. Communication on such topics can contribute significantly to public health [51].

However, the focus of veterinary medicine in public health is not limited to zoonoses, because the One Health concept involves all of the aspects of health, including mental health via the human-animal bond phenomenon [52]. This latter aspect gives to the veterinary medicine a further role in public health, through disciplines centred on human-animal bond such as pet therapy. Moreover, the One Health statement amplifies the role of companion animals clinical practice in maintaining and improving human health, because it concerns not only zoonoses but also the owner-pet relationship.

The importance of communication in clinical practice has been widely recognized, even if only recently in veterinary medicine, where effective communication increases client satisfaction and compliance [53,54,55,56,57,58,59,60]. Therefore, this skill has become part of the veterinary education programs in many countries, being as important as other clinical skills [54,61,62,63].

However, in many European countries communication is still poorly considered. This situation may lead to communication errors, failures in meeting client expectations and, eventually, to complaint or malpractice claim as has been observed in human medicine [64]. Recent studies showed that in one of these countries, the large majority of pet owners consider the own pet as family member and this also affected owner responses and attitudes towards the veterinarian [65]. These results confirm the role of communication in improving veterinary practice and promoting clients’ comfort, satisfaction and well-being.

#### 2.3.2. Food-Producing Animals

Communication has a key role also in food-producing animals practice, improving management and animal health, as demonstrated for example in dairy farms [66,67,68].

Communication strategies are essential in promoting dairy herd health and are required to support diseases control programs [69,70]. Indeed, how consultant delivers information affects ideas, beliefs and motivation of the farmer, thus his/her behaviour and actions. [71,72,73]. Therefore, the leading role of communication in dairy herds is due to its power in influencing how farmers apply consultants' advice and, thus, in improving animal health and food safety. It is crucial for public health in a “from field to fork” perspective, because farmer’s actions and management directly influence the quality of meat, milk and milk products.

All these aspects improve also herd sustainability. An effective communication in food-producing area supports development and improves health and innovation, also in emerging countries.

However, communication in veterinary practice is poorly considered in many countries including Italy. In this country, recent studies showed that dairy farmers are not satisfied with their consultants’ communication [74]. This gap in veterinarians' skill represent a problem because an effective farmer-veterinarian communication is essential to improve farm management, herd health, food safety and herd sustainability.

### 2.4. Pharmacology and Drugs

The activity of veterinarians related to pharmacology and drugs is often cross-disciplinary, covering companion and food producing animal practice, food security, and official drugs residue controls. However, the rising importance of prudent use of antibiotics makes this topic suitable to become a specific area under the One Health approach. Indeed, the concern on antimicrobial resistance (AMR) increased in recent years; it is considered a major public health threat, due to misuse of antibiotics in both human and animal therapy. Therefore, it is a cross-disciplinary issue, engaging veterinarians to reduce and monitor antimicrobial usage in livestock [75,76].

Many veterinary organisations promote the responsible and prudent use of antimicrobial agents in animals, such as The World Organisation for Animal Health (OIE) [77]. Moreover, OIE developed standards and guidelines for OIE Member Countries to address the risk of the emergence or spread of resistant bacteria that result from the use of antimicrobial agents in food producing animals [78].

The European Medicines Agency (EMA) implemented the European Surveillance of Veterinary Antimicrobial Consumption (ESVAC) project, to identify possible risk factors that could lead to the development and spread of antimicrobial resistance in animals. The aim was to develop a harmonised approach for the collection and reporting of data on the use of antimicrobial agents in animals from EU Member States. Thus, EMA published reports on sales of veterinary antimicrobial agents in EU/EEA countries [79]. The International Dairy Federation (FIL-IDF) published a Guide to Prudent Use of Antimicrobial Agents in Dairy Production [80] and also the Federation of Veterinarians of Europe (FVE) developed a document on AMR and prudent use of antibiotics in Veterinary Medicine [81]. A specific interest on this topic was shown by U.S. Food and Drug Administration (FDA) and the U.S. Centers for Disease Control and Prevention (CDC) [82,83]. The latter one funded the development of the Antimicrobial Resistance Learning Site (AMRLS), which is a suite of educational materials promoting the prudent use of antimicrobial agents in veterinary practice [84]. Despite the large interest of veterinary sector on AMR, the communication was poorly considered. However, communication in veterinary medicine can promote the prudent use of antimicrobials, being synergic with the initiatives in human medicine. To highlight the problem and to support a rational antimicrobial use, the European Union promoted the European Awareness Day, an health initiative marked annually on 18 November and coordinated by European Center for Diseases Prevention and Control (ECDC). For this event, ECDC provides campaign communication materials for the European national health authorities in order to develop a consistent communication campaign on the prudent use of antibiotics. National campaigns use several communication tools, such as leaflets, poster, videos, websites and social media, that are aimed to both prescribers and the general public. Many communication initiatives take place across Europe, to disseminate messages on risks related to the antibiotic misuse and explaining how to take antibiotics responsibly [85,86,87]. Similar campaigns are promoted also in the United States, Canada and Australia [88,89].

All these programs show the importance of communication in human medicine to contain AMR, but communication in veterinary medicine is equally indispensable to promote a rational use of antibiotics and enhance the collaboration of veterinary organisations with the public health sector to reduce the antimicrobial resistance**.**

### 2.5. Wildlife Fauna and Environment

The One Health concept considers also the health of ecosystem, being health of humans, animals and environment interrelated. Indeed, both animals and humans live in the same environment, sharing air, water and food. If there is a poor health for the environment, there is a poor health for people and animals. The environmental pollution is a health hazards but it is not the only threat, because the health of plants is indispensable for having food [90]. Through an effective and responsible management of the natural resources, the One Health approach protects human and animal health and ensures a safe food supply [36,37]. Veterinary medicine can contribute to the health of the ecosystem, because agricultural contamination may lead to contamination of foods of animal origin. Moreover, these foods can be used as sentinel for monitoring contamination in the environment. For example, pesticide contamination of honey can be related to the contamination source and could reflect the specific pollution of an environment [91]. The use of animal sentinel for health hazards is very helpful to detect and manage more quickly and efficiently shared health risks [92]. Also when considering toxic risk, as shown by the potentials of “One Toxicology”, the protection of domestic and wild animals’ health is relevant to protecting humans [93].

In recent years the emerging diseases gained a leading role in epidemics on a global scale, and zoonoses were dominant [94]. These diseases are currently the main and global threat for human health, and are related to both domestic and wildlife fauna. Therefore, the synergy between medicine and veterinary medicine is indispensable for the research, the surveillance, the control and the communication of global health challenges at the animal-human-ecosystem interface [95]. The result of this cooperation is well known in developing countries, where it already resulted in an improved health care for the local populations [96]. Also in this area of veterinary medicine, coordinated communication efforts make an important contribution to public health.

## 4. Conclusions

In almost all the areas of veterinary activity communication can contribute to human health. It takes many forms and employs several channels, and this variety of communication opportunities represent a challenge for veterinarians. For this reason, communication courses should be included in the curricula of Veterinary Medicine Schools, as it happened in some already in North America.

Veterinarians should improve their communication skills because human and animal health are interrelated, as is health communication in human and veterinary sectors. Both of them cover the same issue (zoonoses, food safety *etc*.) and use the same channels. For example, it is not possible to differentiate between food risk communication in human and veterinary areas, having the same target, using the same strategies and considering the same risks.

Despite the fact that health communication is a recent matter, it has a growing importance in human medicine but it is less considered in veterinary medicine. However, because of the role of veterinary medicine in public health, communication in veterinary area also has the potential to improve public health. Therefore, a One Health approach applies to communication as well, leading to the “One Communication” concept, which is the result of the synergy in communicative efforts both in human and in veterinary medicine. As One Health, One Communication is a strategy for expanding collaborations in health communication and it will enhance health care.
